# Citric Acid in Drug Formulations Causes Pain by Potentiating Acid-Sensing Ion Channel 1

**DOI:** 10.1523/JNEUROSCI.2087-20.2021

**Published:** 2021-05-26

**Authors:** Ya Lan Yang, Ted Weita Lai

**Affiliations:** ^1^Graduate Institute of Biomedical Sciences, China Medical University, Taichung 40402, Taiwan; ^2^Drug Development Center, China Medical University, Taichung 40402, Taiwan; ^3^Translational Medicine Research Center, China Medical University Hospital, Taichung 40402, Taiwan

**Keywords:** acid, ASIC, citrate, citric acid, pain, pharmaceutical formulation

## Abstract

Pain at the injection site is a common complaint of patients receiving therapeutic formulations containing citric acid. Despite the widely acknowledged role of acid-sensing ion channels (ASICs) in acid-related perception, the specific ASIC subtype mediating pain caused by subcutaneous acid injection and the mechanism by which citrate affects this process are less clear. Here, male mice subjected to intraplantar acid injection responded by executing a withdrawal reflex, and this response was abolished by ASIC1 but not ASIC2 knockout. Although intraplantar injection of neutral citrate solution did not produce this response, intraplantar injection of acidic citrate solution produced a withdrawal reflex greater than that produced by acidity alone. Consistent with the behavioral data, neutral citrate failed to produce an electrophysiological response in HEK293 cells, which express ASIC1, but acidic citrate produced a whole-cell inward current greater than that produced by acidity alone. Saturating the intracellular solution with citrate had no effect on the potentiating effect of extracellular citrate, suggesting that citrate acted extracellularly to potentiate ASIC1. Moreover, exposure to citrate immediately before acid stimulation failed to potentiate ASIC1 currents, which ruled out the involvement of a metabotropic receptor gated by a citrate metabolite. Finally, removal of calcium ions from the extracellular solution mimicked the potentiating effect of citrate and prevented citrate from further potentiating ASIC1. Our data demonstrate that ASIC1 is necessary for the nociceptive response caused by subcutaneous acid infusion and that neutral citrate, despite not inducing ASIC1 currents or nociceptive behavior on its own, potentiates acid nociception by removing the inhibitory effect of extracellular calcium ions on ASIC1.

**SIGNIFICANCE STATEMENT** Citric acid is a common ingredient used in pharmaceutical formulations. Despite the widespread clinical use of these formulations, it remains unclear how citric acid causes pain when injected into patients. We identified ASIC1 as the key receptor used to detect injection-site pain caused by acid, and we showed that neutral citrate does not stimulate ASIC1; instead, citrate substantially potentiates ASIC1 activation when injected simultaneously with acid. In addition, we demonstrated that citrate potentiates ASIC1 by removing the inhibitory action of calcium on the extracellular side of the receptor. Given that injection-site pain is the primary complaint of patients receiving citrate-containing medical products, our data provide mechanistic insight into a common medical complaint and suggest a means of avoiding injection pain.

## Introduction

Acid causes pain when injected subcutaneously or intramuscularly into human subjects (Steen and Reeh, 1993; Steen et al., 1995; Issberner et al., 1996; Ugawa et al., 2002; Jones et al., 2004), and pharmaceutical formulations containing citrate (10–25 mm), widely used for its pH buffering, calcium chelating, and antioxidant properties, cause more pain than citrate-free formulations carrying the same therapeutic agents (Frenken et al., 1993; Veys et al., 1998; Yu et al., 1998; Laursen et al., 2006). As a result, pain at the injection site is a major complaint of patients receiving injections of the current best-selling pharmaceutical product adalimumab (Humira, AbbVie), which has a citrate-buffered acidic formulation, pH 5.2, for the treatment of rheumatoid arthritis and Crohn's disease (Furst et al., 2003; Keystone et al., 2004; van de Putte et al., 2004; Nash et al., 2016). To address this unfavorable property, a citrate-free formulation (Humira Citrate-free) recently became commercially available. Moreover, tissue acidosis has been thought to contribute to pain sensation caused by ischemia, inflammation, and surgical incision (Issberner et al., 1996; Benson et al., 1999; Pan et al., 1999; Woo et al., 2004), and citrate levels have been associated with episodes of angina pectoris (Bagger et al., 1981); therefore, understanding the mechanism by which acid or citrate causes pain can have broad clinical implications for understanding pain beyond therapeutic injections. Mammalian cells sense tissue acidification primarily through proton-gated ion channels, including the acid-sensing ion channel (ASIC) family (Price et al., 1996; Waldmann et al., 1996, 1997a,b; García-Añoveros et al., 1997) and transient receptor potential cation channel subfamily V member 1 (TrpV1; Caterina et al., 1997; Tominaga et al., 1998). ASIC is further categorized into subtypes encoded by four separate genes that encode ASIC1a (García-Añoveros et al., 1997; Waldmann et al., 1997a) and its splice variant ASIC1b (Chen et al., 1998; Bässler et al., 2001), ASIC2a (Price et al., 1996; Waldmann et al., 1996; García-Añoveros et al., 1997) and its splice variant ASIC2b (Lingueglia et al., 1997), ASIC3 (Waldmann et al., 1997b), and ASIC4 (Akopian et al., 2000; Gründer et al., 2000); among these ASIC subtypes, all but ASIC2b and ASIC4 form proton-gated ion channels on their own (Lingueglia et al., 1997; Akopian et al., 2000; Gründer et al., 2000). Although it is widely believed that these proton-gated ion channels, which are expressed on the neurite terminals of nociceptors, are likely initiators of acid-related sensory transduction, the receptor subtype that mediates acute pain caused by subcutaneous acid injection remains unclear, as does the contribution of citrate to this painful sensation.

In this study, we asked whether neutral citrate at a concentration resembling pharmaceutical products causes acid-like pain when injected subcutaneously in mice and whether it affects pain caused by intraplantar injection of acid at a pH resembling pharmaceutical products. We found that intraplantar acid injection causes a rapid withdrawal reflex in mice, a response that can be attenuated by coinjection of the ASIC inhibitor amiloride. Although neutral citrate failed to produce such a response, it substantially augmented the nociceptive response caused by the acid injection. To investigate this phenomenon further, we identified ASIC1 as the receptor subtype required for this type of pain perception *in vivo*, and, in a cell line natively expressing human ASIC1, we further investigated the mechanism by which citrate contributes to acid-induced nociception by studying its pharmacological effect on ASIC1 electrophysiology *in vitro*.

## Materials and Methods

### 

#### 

##### Mice

Male C57BL/6 mice (6–7 weeks old, 20–30 × g) were used in most experiments. Mice lacking ASIC1 (B6.129-*Asic1^tm1Wsh^*/J, stock #013733; Wemmie et al., 2002), ASIC2 (B6.129-*Asic2^tm1Wsh^*/J, stock #013126; Price et al., 2000), and TrpV1 (B6.129×1-*Trpv1^tm1Jul^*/J, stock #003770; Caterina et al., 2000) were purchased from The Jackson Laboratory. The mice were provided food and water *ad libitum* before the experiment, and all experiments involving animals followed the Institutional Guidelines of the China Medical University for the Care and Use of Experimental Animals and were approved by the university's Institutional Animal Care and Use Committee (Protocol No. 2016–213-2 and 2020–401).

##### Cell culture

HEK293 cells (catalog #CRL-1573, American Type Culture Collection) were used in the electrophysiological experiments. The cells were cultured in DMEM, supplemented with 10% fetal bovine serum (FBS) in a CO_2_ incubator (catalog #310TS, Thermo Scientific) until used for experiments.

##### Drugs and chemicals

Sodium citrate tribasic dihydrate and capsaicin were purchased from Sigma-Aldrich (catalog #S4641 and #M2028, respectively), amiloride was purchased from Alomone Labs (catalog #A-140), 10% neutral buffered formalin was purchased from TONYAR BIOTECH. INC. (catalog #50-00-0), and glacial acetic acid was purchased from J.T.Baker (catalog #9508-03).

##### Nociceptive model—withdrawal test

The withdrawal test was designed to assess pain experienced at the moment of subcutaneous injection. To minimize voluntary movements, each mouse was lightly anesthetized by 1.5% isoflurane (carried by air) induction in a gas chamber, and anesthesia was maintained with a gas mask. This dose of isoflurane sedates the mouse sufficiently to abolish the righting reflex but maintains the mouse's withdrawal reflex in response to a toe pinch. Paired test solutions were injected into opposite hindpaws one at a time (10 µl/injection), and the relative withdrawal response between each pair of injections was recorded. Specifically, (+), (=), or (−) was recorded when the response in one limb was greater than, equal to, or less than the response in the other limb, respectively. In addition, (o) was recorded when there was no withdrawal response. The order in which each of the paired treatments was injected was initially randomized and then systemically reversed, so each of the paired treatments had an equal chance of being injected first. The investigators performing the experiments were blinded to the treatments. For animals of different genotypes, genotyping was conducted after the completion of the experiments, so the investigators were blind to the genotypes of the animals at the time of the experiment.

##### Nociceptive models—paw-licking and writhing tests

Conventional nociceptive tests based on formalin-induced paw licking (Hunskaar et al., 1985) and acetic-acid-induced writhing (Witkin et al., 1961; Collier et al., 1968) were additionally used to assess acute chemical-induced pain responses. To assess paw-licking behavior, each mouse received an injection of a test solution into a hindpaw (25 µl/injection) under brief isoflurane-induced anesthesia, and the duration of its paw-licking behavior was recorded for a period of 30 min. To assess writhing behavior, each mouse received an injection of a test solution into its peritoneal cavity (10 µl/g) under brief isoflurane-induced anesthesia, and the numbers of abdominal contractions or body contortions over a period of 10 min were recorded. The investigators performing the experiments were blinded to the treatments or animal genotypes.

##### Electrophysiology—human ASIC1

Whole-cell patch-clamp recording of native human ASIC1 current was performed as previously described (Yang and Lai, 2020). In brief, HEK293 cells were perfused in a bath solution containing the following (in mm): 152 NaCl, 2.5 KCl, 2 CaCl_2_, 1 MgCl_2_, and 5 HEPES (the pH and osmolarity were 7.4 and 300 ± 10 mOsm, respectively) and patched with glass pipettes (tip resistance of 4–6 MΩ) filled with intracellular solution (ICS) containing the following (in mm): 10 NaCl, 120 KCl, 0.5 CaCl2, 2 MgCl2, 5 EGTA, 10 HEPES, and 2 Mg-ATP (the pH and osmolarity were 7.2 and 310 mOsm, respectively). The membrane potential was held at −60 mV in all experiments, and each stimulus consisted of a 3 s switch (at 30 s intervals) from the bath solution to the test solution, preceded by a voltage step of −10 mV to detect abrupt changes in membrane capacitance and access resistance. Only one cell was recorded for each independent culture (neighboring cells from the same culture were not recorded), so the sample size represents both the number of independent cultures as well as the number of individual cells recorded.

##### Electrophysiology—primary sensory neurons

Native ASIC current was recorded from primary sensory neurons collected from dorsal root ganglion (DRG) of isoflurane-anesthetized adult male C57BL/6 mice (6–7 weeks old, 20–30 × g). The isolated DRG was kept on ice-cold Ca^2+^/Mg^2+^-free HBSS, dissociated with 0.1% collagenase and 0.25% trypsin, and cultured in DMEM/F12 supplemented with 10% FBS. On the day of recording, the neurons were bathed in extracellular solution, which contained the following (in mm): 145 NaCl, 5 KCl, 2 MgCl_2_, 2 CaCl_2_, 10 HEPES (or 10 MES for pH 5.5), and was pH adjusted to 7.4 or 5.5 with NaOH or HCl, respectively, and patched with glass pipettes (tip resistance of 2-3 MΩ) filled with ICS, which contained the following (in mm): 10 NaCl, 120 KCl, 2 MgCl_2_, 0.5 CaCl_2_, 5 EGTA, 10 HEPES, 2 Mg-ATP, and its pH and osmolarity were adjusted to 7.2 with KOH and 310 mOsm with sucrose, respectively. The membrane potential was held at −60 mV in all experiments, and each stimulus consisted of a 3 s switch (at 30 s intervals) from the bath solution to the test solution and was preceded by a voltage step of −10 mV to detect abrupt changes in membrane capacitance and access resistance.

##### Data presentation and analysis

Animal behavior data are shown as individual data points, and cellular electrophysiology data are presented as the mean ± SEM. Significant differences among groups are noted when the *p* value is <0.05. Nonparametric comparisons of withdrawal reflex intensity between paired injections were made using a two-tailed Mann–Whitney *U* test, in which a score of 1 (+) or 0 (o) was assigned for the presence or absence of a response, respectively; additionally, when these comparisons were made between paired injections of test solutions that both consistently produce a response, a score of 1 (+), 0 (=), or −1 (−) was assigned when the response in one limb was greater than, equal to, or less than the response in the other limb, respectively. Nonparametric comparisons among multiple groups of animals were made by one-way ANOVA with a *post hoc* Dunnett's test, in which animals with a response were given a score of 1 (+), and animals without a response were given a score of 0 (o). Parametric comparisons between two means were made by a two-tailed Student's *t* test, those between multiple means by one-way ANOVA with a *post hoc* Tukey's multiple comparisons test, and those between multiple means with two matching factors were made by two-way repeated-measures ANOVA with a *post hoc* Tukey's multiple comparisons test.

## Results

### Citrate potentiates but does not induce acid pain

**Figure 1.**
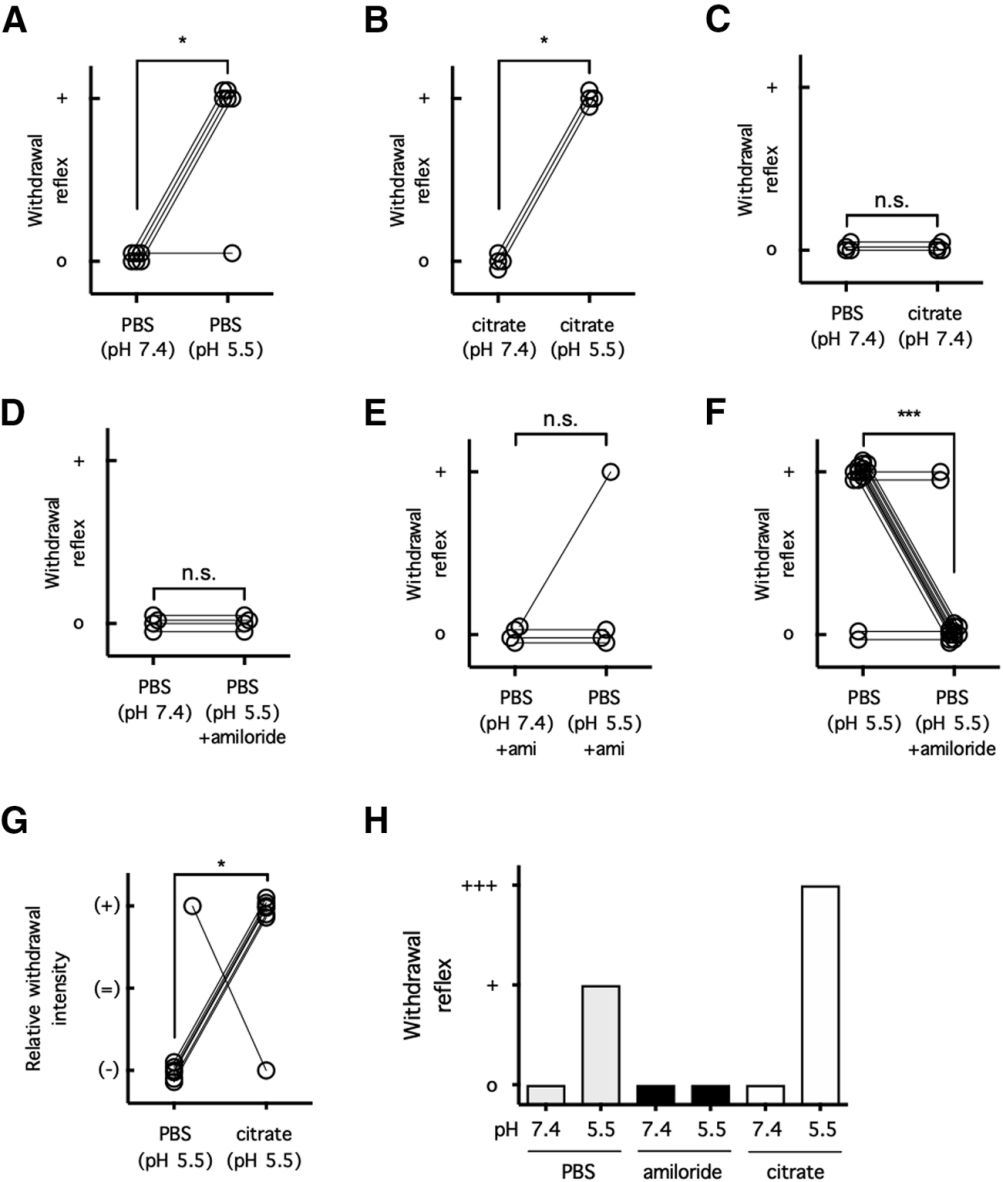
Citrate potentiates ASIC-dependent pain behavior. ***A–C***, Each mouse was injected with either PBS, pH 7.4 (***A, C***), or citrate, pH 7.4 (***B***) in one hindpaw and then PBS, pH 5.5 (***A***); or citrate, pH 5.5 (***B***); or citrate, pH 7.4 (***C***), in the other hindpaw, or vice versa. The presence (+) or absence (o) of a withdrawal reflex was recorded; *n* = 4–6 per group, n.s. *p* > 0.05, *p < 0.05, two-tailed Mann–Whitney *U* test, in which (+) was ranked as 1 and (o) was ranked as 0. ***D–F***, Each mouse was injected in either hindpaw with PBS, pH 7.4 or 5.5, as described for ***A–C***, with or without amiloride (100 μm). The presence (+) or absence (o) of a withdrawal reflex was recorded; *n* = 4–14 per group, n.s. *p* > 0.05, ***p < 0.001, two-tailed Mann–Whitney *U* test, in which (+) was ranked as 1 and (o) was ranked as 0. ***G***, Each mouse was injected with PBS, pH 5.5, in one hindpaw and then citrate, pH 5.5 (25 mm), in the other hindpaw, or vice versa. The relative withdrawal intensity between the two hindpaws was compared and recorded: (+) for greater, (=) for equivalent, and (−) for lesser withdrawal response; *n* = 7 per group, *p < 0.05, two-tailed Mann–Whitney *U* test, in which (+) was ranked as +1, (=) was ranked as 0, and (−) was ranked as −1. ***H***, Summary of the results from Figure 1. n.s., Not significant.

To determine whether citrate at neutral or acidic pH produces a nociceptive response at the moment of subcutaneous injection and how this response compares to that produced by PBS-buffered acid solution (PBAS), mice were subjected to intraplantar injections of neutral, pH 7.4, or acidic, pH 5.5, solutions buffered with PBS or concentrated citrate (25 mm; Fig. 1*A,C*). PBAS, pH 5.5, or acidic citrate solution both produced a rapid withdrawal reflex when injected into mice (*p* = 0.0152 for PBS; *p* = 0.0286 for citrate; Fig. 1*A,B*). Consistent with the role of ASICs in this response, the withdrawal reflex triggered by PBAS was mitigated when the broad-spectrum ASIC inhibitor amiloride was coadministered (PBAS with amiloride *p* > 0.9999 compared with neutral PBS; *p* > 0.9999 compared with neutral PBS with amiloride; *p* = 0.0004 compared with PBAS alone; Fig. 1*D*–*F*). As a negative control, injection of neutral PBS did not produce the rapid withdrawal reflex mentioned above (Fig. 1*A,C*). Somewhat unexpectedly, however, injection of concentrated citrate (25 mm) at a neutral pH value, pH 7.4, also did not produce this nociceptive response (*p* > 0.9999 compared with neutral PBS; Fig. 1*C*). To compare the relative pain intensity triggered by PBAS, pH 5.5, versus acidic citrate, pH 5.5, mice were subjected to intraplantar injections of either solution into one hindpaw followed by the other solution into the other hindpaw (Fig. 1*G*). Although neutral citrate, pH 7.4, did not cause an acid pain response, the nociceptive response produced by acidic citrate, pH 5.5, was consistently much more intense than that induced by PBAS (*p* = 0.0291, pH 5.5; Fig. 1*G*). These data altogether suggest that although citrate per se does not induce ASIC-mediated pain, it strongly potentiates ASIC-mediated pain caused by an acidic stimulus (Fig. 1*H*).

### ASIC1 is the key receptor subtype involved in acute acid pain

**Figure 2.**
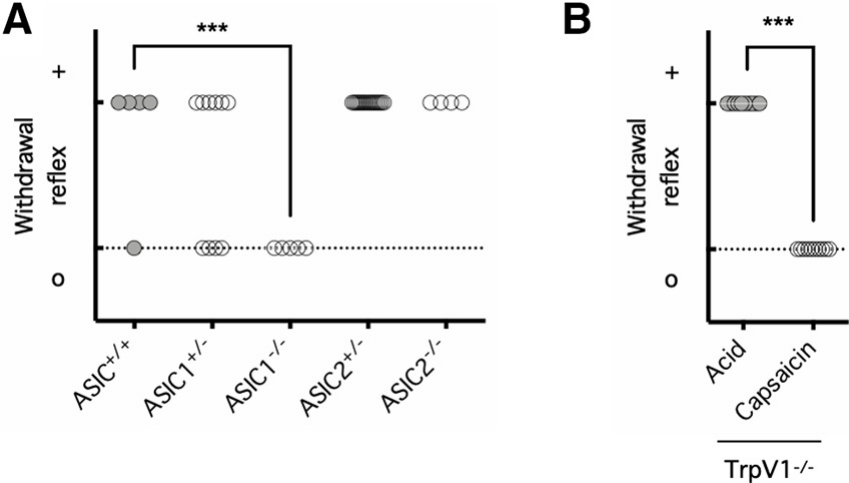
Pain caused by subcutaneous acid is mediated by ASIC1. ***A***, Mice with intact ASIC1 and ASIC2 (*ASIC*^+/+^), hemizygous ASIC1 deletion (*ASIC1*^+/−^), homozygous ASIC1 deletion (*ASIC1*^−/−^), hemizygous ASIC2 deletion (*ASIC2*^+/−^), or homozygous ASIC2 deletion (*ASIC2*^−/−^) were injected with PBS, pH 5.5, into one hindpaw. The presence (+) or absence (o) of a withdrawal reflex was recorded; *n* = 5–17 per group, ***p < 0.001, one-way ANOVA with a *post hoc* Dunnett's test, in which (+) was ranked as 1 and (o) was ranked as 0. ***B***, Mice lacking TrpV1 (*TrpV1*^−/−^) were injected with PBS, pH 5.5, or capsaicin (1 µg) into one hindpaw. The presence (+) or absence (o) of a withdrawal reflex was recorded; *n* = 10 per group, ***p < 0.001, two-tailed Mann–Whitney *U* test, in which (+) was ranked as 1 and (o) was ranked as 0.

There is now ample evidence that the ASIC family of excitatory ion channels is required for acute and chronic acid-related pain, but it is less clear which ASIC subtype is required for which type of acid-related pain. Given the agonistic effect of amiloride on ASIC3 (Yagi et al., 2006; Li et al., 2011) and the lack of nociceptive response on amiloride injection at neutral pH (Fig. 1*E*), we reasoned that ASIC3 is unlikely to be the receptor subtype for PBAS-induced withdrawal reflex. On the other hand, given the antagonistic effect of amiloride on ASIC1 and ASIC2 and the inhibition of the PBAS-induced withdrawal reflex by amiloride (Fig. 1*D–F*), we next asked whether either ASIC1, ASIC2, or both might be involved in this nociceptive response. To investigate this possibility, mice partially or completely lacking ASIC1 (*ASIC1*^+/−^; *ASIC1*^−/−^) or ASIC2 (*ASIC2*^+/−^; *ASIC2*^−/−^) or control mice with intact ASIC1 and 2 receptors (*ASIC*^+/+^) were subjected to intraplantar injections of PBAS, pH 5.5 (Fig. 2*A*). Impressively, *ASIC1*^−/−^ mice consistently failed to demonstrate any acute nociceptive response on intraplantar PBAS injection (*p* = 0.5250 for *ASIC1*^+/−^ mice and *p* = 0.0005 for *ASIC1*^−/−^ mice, compared with *ASIC*^+/+^ mice; Fig. 2*A*). In marked contrast, *ASIC2*^+/−^ and *ASIC2*^−/−^ mice consistently demonstrated an intact nociceptive response on intraplantar PBAS injection (*p* = 0.4639 for *ASIC2*^+/−^ mice and *p* = 0.6801 for *ASIC2*^−/−^ mice, compared with *ASIC*^+/+^ mice; Fig. 2*A*). In line with early evidence that ASIC is solely responsible for subcutaneous pain caused by mild acidity, whereas both ASIC and TrpV1 contribute to subcutaneous pain caused by severe acidity (Ugawa et al., 2002), mice lacking TrpV1 (*TrpV1*^−/−^) demonstrated an intact nociceptive response on intraplantar injection of PBAS, which mimicked pharmaceutical formulations to be only mildly acidic, pH 5.5, but not on intraplantar injection of TrpV1 agonist capsaicin (*p* < 0.0001; Fig. 2*B*). These data demonstrate that ASIC1 and not ASIC2 is the receptor subtype mediating the pain that triggers the withdrawal reflex on intraplantar acid injection.

### Citrate potentiates but does not stimulate ASIC1

**Figure 3.**
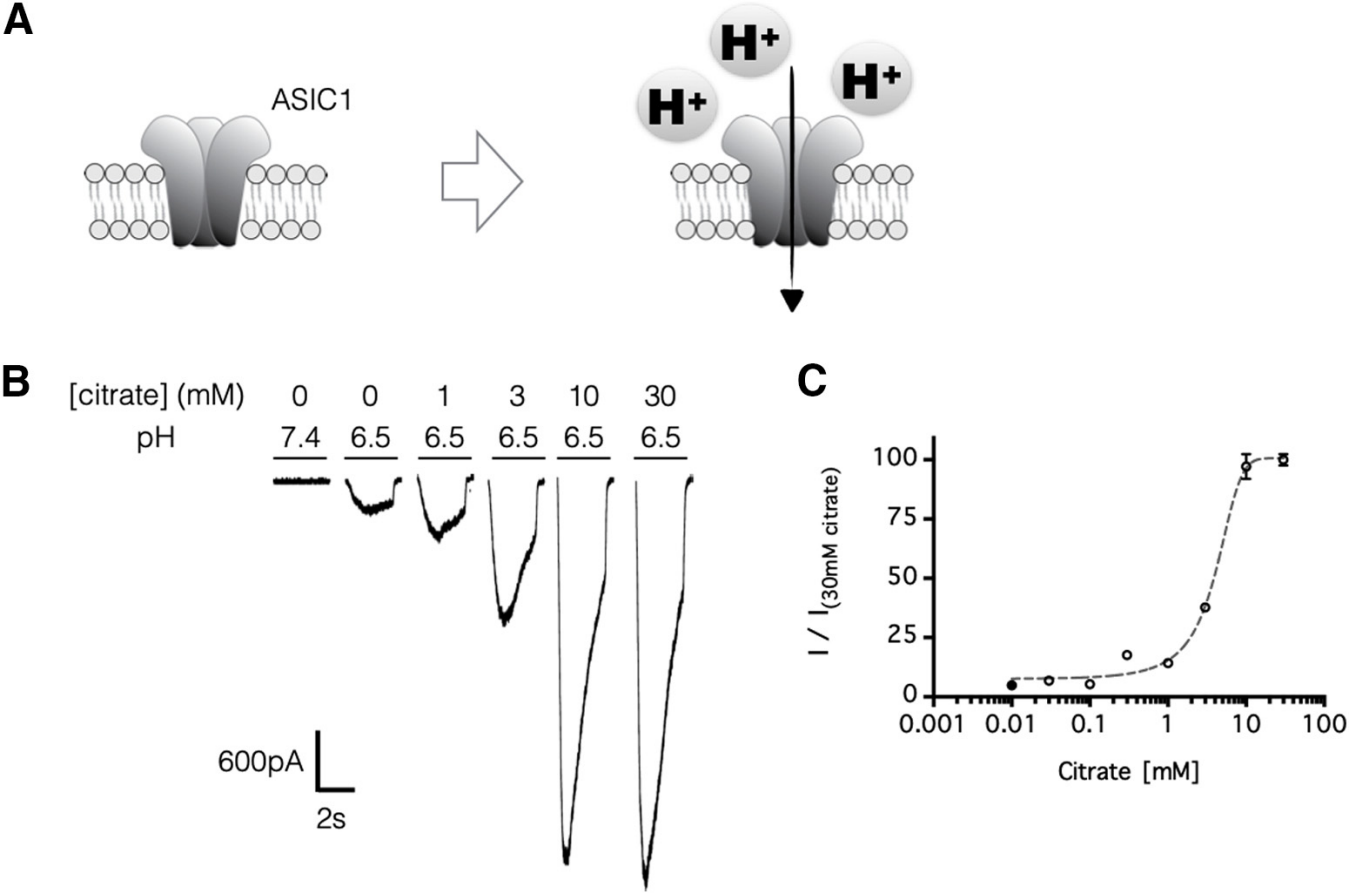
Citrate potentiates ASIC1 currents in a concentration-dependent manner. ***A***, Schematics: ASIC1 generates an inward current in response to increased extracellular protons. ***B***, ***C***, ASIC1-mediated currents were recorded in HEK293 cells. The cells were perfused in a bath solution, pH 7.4, containing no citrate, and each stimulus consisted of a 3 s exposure (at 30 s intervals) to a test solution, pH 6.5, except where pH 7.4 is indicated, that contained 0–30 mm citrate. In ***C***, all test solutions had a pH of 6.5, and the concentrations of citrate in the test solutions are indicated on the *x*-axis; *n* = 4–5 independent HEK293 cultures, and error bars indicate the mean ± SEM.

**Figure 4.**
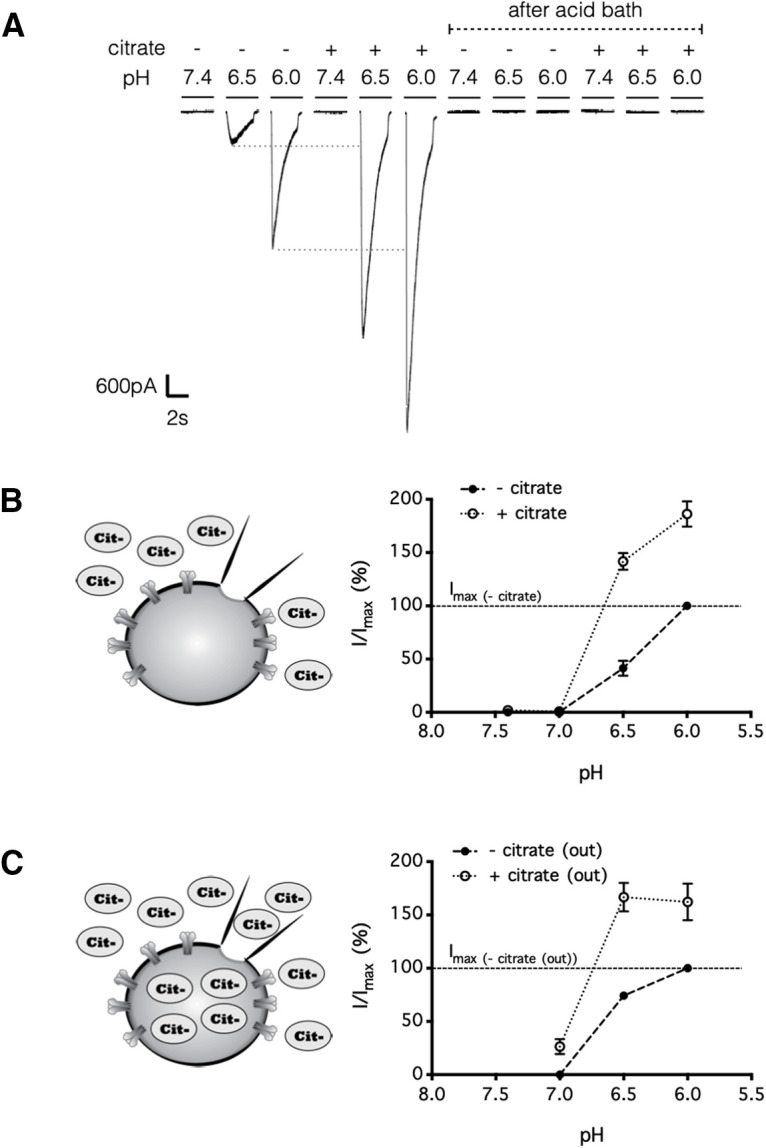
Citrate targets the extracellular site of ASIC1 to potentiate acid-induced currents. ***A***, ***B***, Extracellular citrate increased efficacy of ASIC1 currents in HEK293 cells. The cells were perfused in a bath solution, pH 7.4, containing no citrate, and each stimulus consisted of a 3 s exposure (at 30 s intervals) to a test solution, pH 6.0–7.4, with or without citrate (25 mm). In ***A***, after changing to an acid bath solution, pH 6.5, inward current can no longer be triggered by the same set of test solutions. In ***B***, bath pH was maintained at pH 7.4; *n* = 6 independent HEK293 cultures. ***C***, Similar to ***B***, except that the intracellular solution was preloaded with 25 mm citrate before recording; *n* = 8 independent HEK293 cultures. In ***B*** and ***C***, error bars indicate the mean ± SEM.

The lack of a PBAS-induced withdrawal reflex with amiloride-mediated inhibition (Fig. 1*E–G*) or ASIC1 gene knockout (Fig. 2*A*) suggested that acute pain caused by intraplantar acid injection was mediated by ASIC1. Nevertheless, this finding did not explain how citrate could potentiate ASIC1-mediated pain behavior (Fig. 1*H*). To examine this further, we investigated the pharmacological effect of citrate on the electrophysiological properties of ASIC1 (Fig. 3*A*). We previously reported that HEK293 cells express native human ASIC1a and ASIC1b homologues at proportions similar to what is found in human primary sensory neurons, making this cell line suitable for studying the electrophysiology of ASIC1-mediated pain (Yang and Lai, 2020). Here, in line with the potentiating effect of citrate on ASIC-mediated nociceptive behavior, citrate (1–30 mm) markedly potentiated HEK293 ASIC1 currents in a concentration-dependent manner, with an EC_50_ of 3–5 mm (Fig. 3*B,C*). Moreover, consistent with the *in vivo* data showing that citrate at neutral pH did not induce ASIC-mediated pain (Fig. 1*B,C*), citrate at pH 7.4 did not induce any inward current in HEK293 cells (Fig. 4*A,B*). Therefore, our data demonstrated that citrate markedly increases the efficacy (maximum response) but has little or no effect on the potency (EC_50_) of ASIC1 channels *in vitro* (Fig. 4*A,B*), which corresponds to its effect on pain behavior *in vivo*.

**Figure 5.**
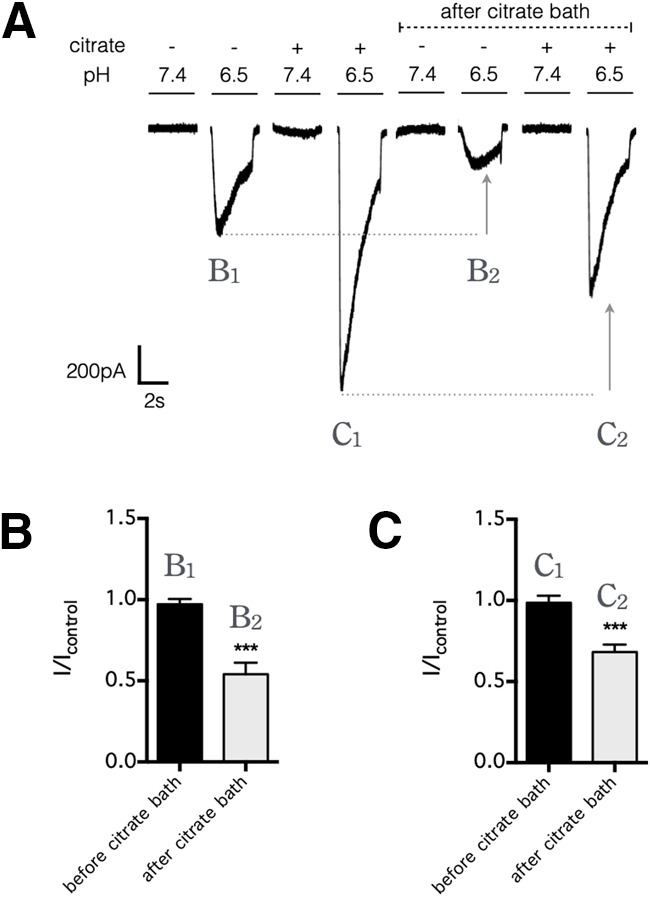
Citrate potentiates ASIC1 current only when applied concurrently with each stimulus. ***A***, HEK293 cells were initially perfused in a bath solution, pH 7.4, without citrate, and each stimulus consisted of a 3 s exposure (at 30 s intervals) to a test solution, pH 7.4 or 6.5, with or without citrate (25 mm). Thereafter, the cells were perfused in a bath solution, pH 7.4, that contained citrate (25 mm); after the citrate bath, the same set of test solutions now produce smaller inward currents. The result indicates that ASIC1-mediated current was potentiated by the concurrent presence of citrate during the pH drop (B_1_ vs C_1_ or B_2_ vs C_2_) but was depressed (as indicated by arrows) by a preexposure to citrate before the pH drop (B_1_ vs B_2_ or C_1_ vs C_2_). ***B***, ***C***, Summarized results from ***A***, comparing current amplitudes of B_1_ to B_2_ (***B***) and C_1_ to C_2_ (***C***); error bars indicate the mean ± SEM; *n* = 6 independent HEK293 cultures. ***p < 0.001, two-tailed Student's *t* test.

**Figure 6.**
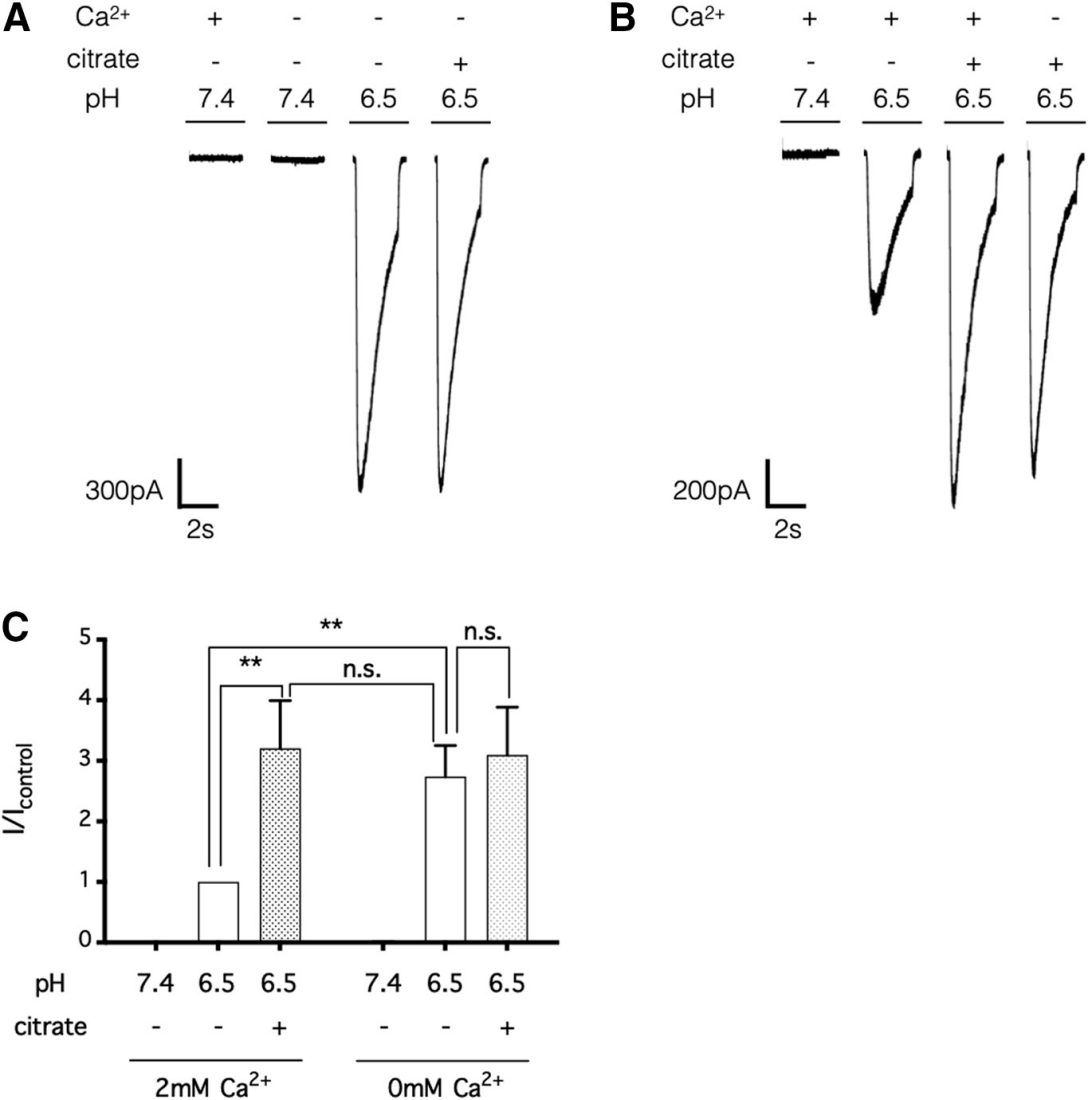
Citrate potentiates the ASIC1 current by chelating extracellular calcium ions. ***A***, ***B***, HEK293 cells were perfused in a bath solution, pH 7.4, containing 2 mm Ca^2+^ and no citrate, and each stimulus consisted of a 3 s exposure (at 30 s intervals) to a test solution, pH 7.4 or 6.5, with or without citrate (25 mm) and/or Ca^2+^ (2 mm). The results indicate that citrate failed to potentiate the ASIC1-mediated current triggered by a Ca^2+^-free test solution, pH 6.5 (***A***), and that Ca^2+^ removal failed to further potentiate the ASIC1-mediated current triggered by a test solution containing citrate (25 mm; ***B***). ***C***, Summarized results from ***A*** and ***B***; error bars indicate the mean ± SEM; *n* = 5 independent HEK293 cultures; n.s. *p* > 0.05, **p < 0.01, two-way repeated measures analysis of variance (matching recordings from the same cell) with a *post hoc* Tukey's test. n.s., Not significant.

Knowing that citrate alone did not activate ASIC1 (Fig. 4*A,B*), we next conducted experiments to investigate how citrate potentiated ASIC1 currents. First, preloading the cells with an equal concentration of intracellular citrate did not prevent the potentiation of ASIC1 current by extracellularly applied citrate (Fig. 4*C*), demonstrating that citrate exerted its effect by acting on the extracellular rather than the intracellular domain of ASIC1. Second, citrate depressed rather than potentiated ASIC1 currents when administered before rather than concurrently with acidic pH (*p* = 0.0002 for acid stimuli after bath exposure to neutral citrate, *p* = 0.0008 for acidic citrate stimuli after bath exposure to neutral citrate; Fig. 5*A–C*). Thus, the effect of extracellular citrate on ASIC1 was likely direct, rather than requiring an intermediate second messenger generated, for instance, from metabotropic receptors gated by citrate analogs (He et al., 2004). Third, removal of Ca^2+^, a known inhibitor of ASIC1 (Paukert et al., 2004; Zhang et al., 2006; Zuo et al., 2018), from the extracellular solution also potentiated ASIC1 currents in HEK cells (*p* < 0.01, compared with acid stimuli in Ca^2+^-intact extracellular solution), and in this situation, citrate failed to further potentiate the response (*p* > 0.05, compared with acid stimuli in Ca^2+^-free extracellular solution; Fig. 6*A–C*). Together, our electrophysiological data demonstrated that concurrent citrate treatment potentiated ASIC1 by chelation of Ca^2+^ from the extracellular domain of the ion channel.

### Citrate also potentiates ASIC subtypes not required for subcutaneous acid pain

**Figure 7.**
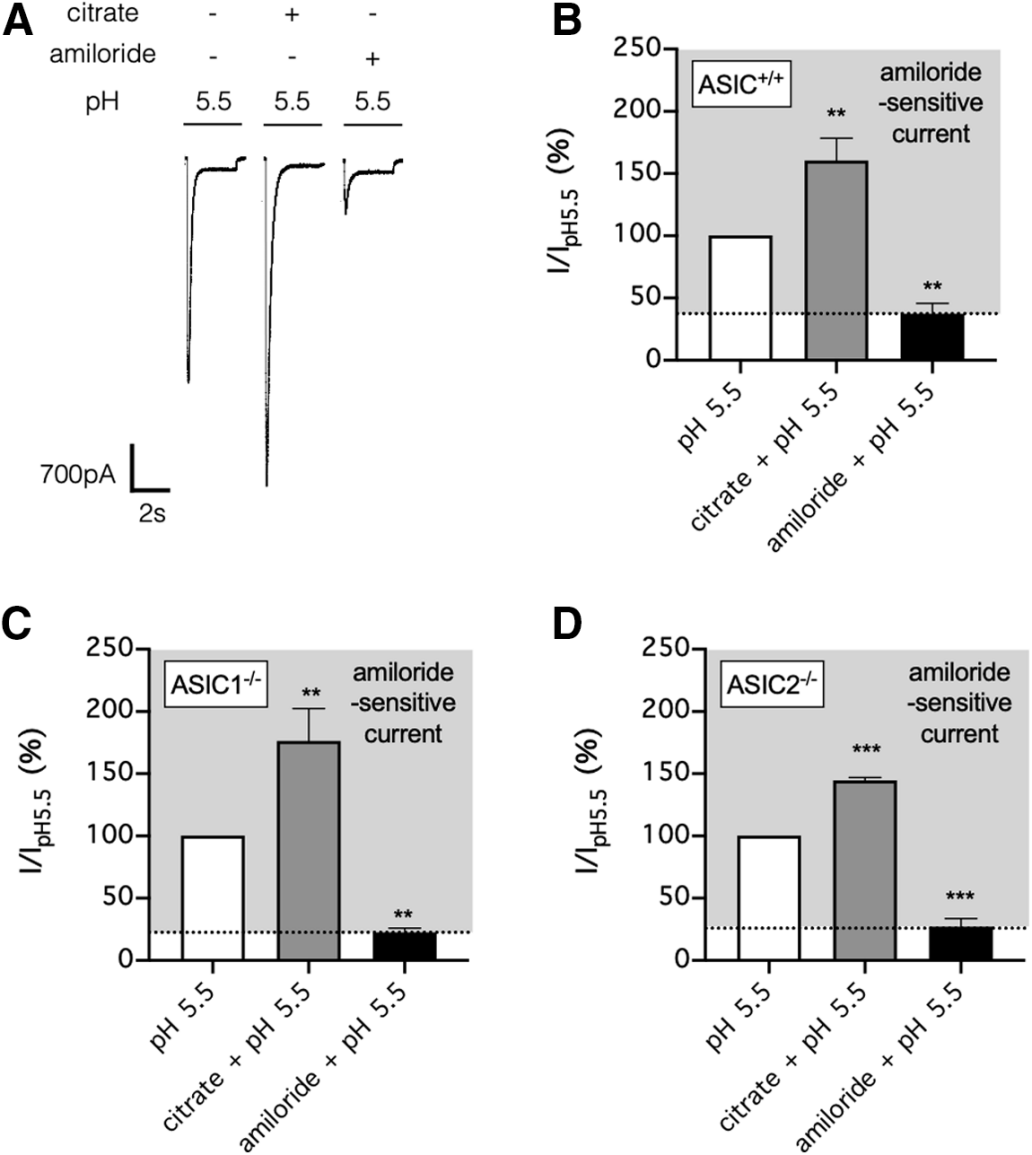
Citrate also potentiates ASIC lacking ASIC1 subunit in mouse primary sensory neurons. ***A***, Primary sensory neurons isolated from the mouse dorsal root ganglia were perfused in a bath solution, pH 7.4, containing no citrate, and each stimulus consisted of a 3 s exposure (at 30 s intervals) to a test solution, pH 5.5, with or without citrate (25 mm) or amiloride (100 μm). ***B***, Summarized result from ***A***. ***C***, ***D***, Similar to ***B***, except the primary sensory neurons were from mice lacking ASIC1 (*ASIC1*^−/−^; ***C***) and ASIC2 (*ASIC2*^−/−^; ***D***), respectively. In ***B–D***, error bars indicate the mean ± SEM; *n* = 5 per group, **p < 0.01, ***p < 0.001, one-way ANOVA with a *post hoc* Tukey's test.

The primary sensory neurons express many different acid-sensitive receptors and ion channels, with many possible subunit combinations. Complementary to our data showing that ASIC1 but not ASIC2 subunit of the ASIC family is required for pain caused by subcutaneous injection of acid (at a pH relevant for pharmaceutical formulations; Fig. 2*A*), previous studies have noted the involvement of other ASIC subtypes of the primary sensory neurons in other types of sensory perceptions and/or physiological responses (Price et al., 2000, 2001; Lin et al., 2016). Therefore, we next ask whether citrate potentiates ASIC natively expressed by primary sensory neurons in wild-type mice with intact pain perception and/or in *ASIC1*^−/−^ mice lacking subcutaneous acid pain perception (Fig. 7*A–D*). As with ASIC1 expressed in HEK cells, citrate potentiated amiloride-sensitive acid current in primary sensory neurons isolated from wild-type mouse dorsal root ganglia (*p* = 0.0074, for comparison of acid current with or without citrate; *p* = 0.0059, for comparison of acid current with or without amiloride; (Fig. 7*A,B*). Notably, in primary sensory neurons from *ASIC1*^−/−^ mice, citrate also potentiated amiloride-sensitive acid current (*p* = 0.0097, for comparison of acid current with or without citrate; *p* = 0.0090, for comparison of acid current with or without amiloride; Fig. 7*C*). Likewise, in primary sensory neurons from *ASIC2*^−/−^ mice, citrate potentiated amiloride-sensitive acid current (*p* < 0.0001, for comparison of acid current with or without citrate; *p* < 0.0001, for comparison of acid current with or without amiloride; Fig. 7*D*). Together, our data suggest that citrate can potentiate ASIC subtypes involved in other sensory modalities or physiological responses in addition to its effect on pain perception mediated by ASIC1.

### ASIC1 is not required for acid-induced paw-licking and writhing responses

**Figure 8.**
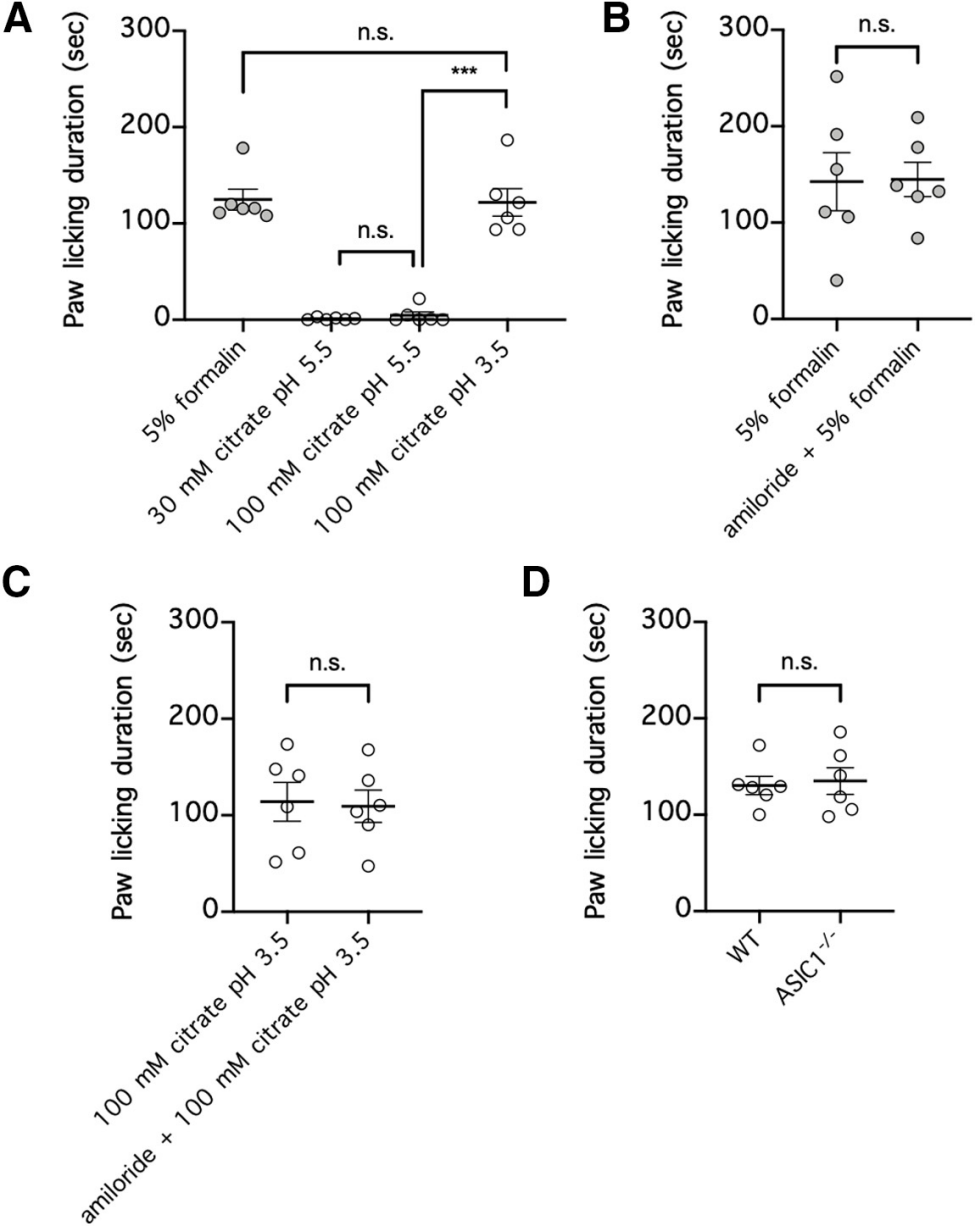
Concentrated citric acid at pH 3.5 but not at pH 5.5 induces paw-licking behavior that is ASIC1 independent. ***A***, Each mouse was injected with either 5% formalin; 30 mm citrate, pH 5.5; or 100 mm citrate, pH 5.5 or 3.5, into one hindpaw, and the duration of paw licking over a period of 30 min was recorded; *n* = 6 per group, n.s. *p* > 0.05, ***p < 0.001, one-way ANOVA with a *post hoc* Tukey's test. ***B***, ***C***, Each mouse was injected with either 5% formalin, with or without amiloride (100 μm; ***B***) or 100 mm citrate, pH 3.5, with or without amiloride (100 μm; ***C***) into one hindpaw, and the duration of paw licking over a period of 30 min was recorded; *n* = 6 per group, n.s. *p* > 0.05, two-tailed Student's *t* test. ***D***, wild-type mice (WT) or mice with homozygous ASIC1 deletion (*ASIC1*^−/−^) were injected with 100 mm citrate, pH 3.5, into one hindpaw, and the duration of paw licking over a period of 30 min was recorded; *n* = 6 per group, n.s. *p* > 0.05, two-tailed Student's *t* test. n.s., Not significant.

**Figure 9.**
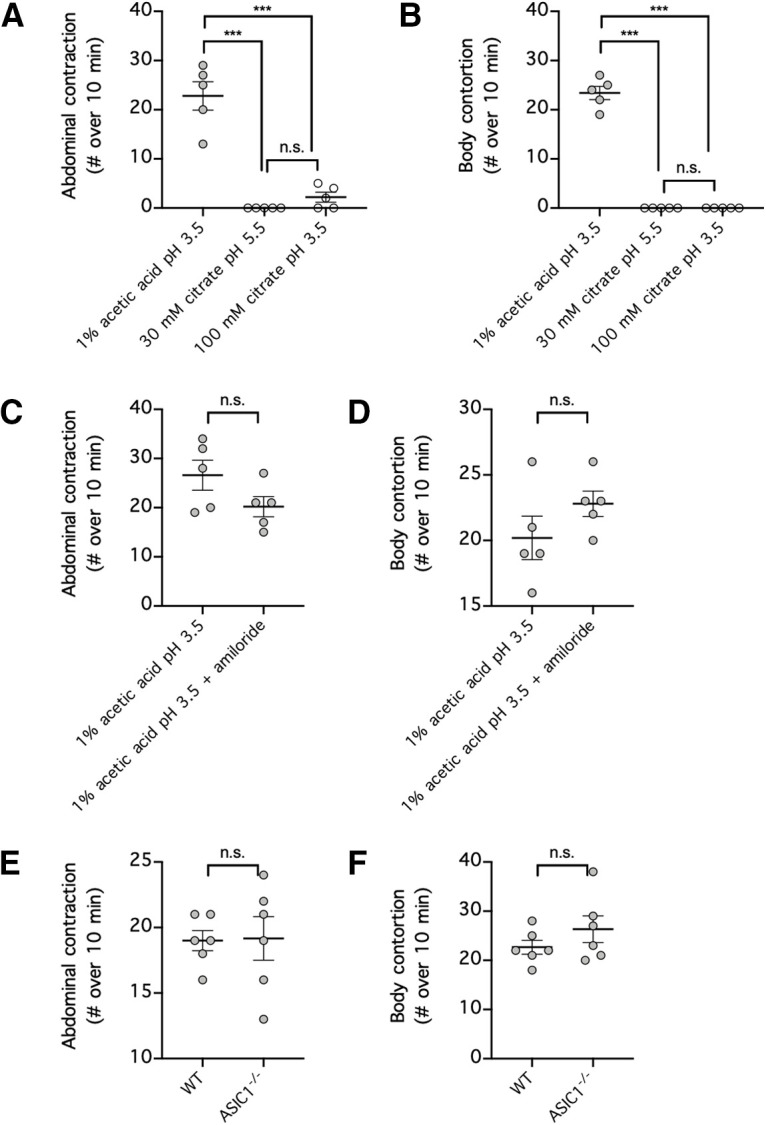
Acetic acid but not citric acid induces writhing behavior that is ASIC1 independent. ***A***, ***B***, Each mouse received intraperitoneal injection of either 1% acetic acid, pH 3.5; 30 mm citric acid, pH 5.5; or 100 mm citric acid, pH 3.5; and the numbers of abdominal contractions (***A***) and body contortions (***B***) over a period of 10 min were recorded; *n* = 5 per group. n.s. *p* > 0.05, ***p < 0.001, one-way ANOVA with a *post hoc* Tukey's test. ***C***, ***D***, Each mouse received intraperitoneal injection of 1% acetic acid, pH 3.5, with or without amiloride (100 μm), and the numbers of abdominal contractions (***C***) and body contortions (***D***) over a period of 10 min were recorded; *n* = 5 per group, n.s. *p* > 0.05, two-tailed Student's *t* test. ***E***, ***F***, wild-type mice (WT) or mice with homozygous ASIC1 deletion (*ASIC1*^−/−^) received intraperitoneal injection of 1% acetic acid, pH 3.5, and the numbers of abdominal contractions (***E***) and body contortions (***F***) over a period of 10 min were recorded; *n* = 6 per group, n.s. *p* > 0.05, two-tailed Student's *t* test. n.s., Not significant.

Among the conventional rodent models of nociception, the formalin-induced paw-licking test and the acetic-acid-induced writhing test are the most widely used for assessing chemical-induced acute pain (Le Bars et al., 2001; González-Cano et al., 2020). Therefore, we also assessed pain responses caused by citric acid in these models and examined whether these responses are also ASIC1 dependent. In the paw-licking test, injection of 5% formalin into a hindpaw readily caused a paw-licking response lasting over 100 s in duration (Fig. 8*A*). Although injection of citric acid resembling the concentration (30 mm) and pH 5.5 used in pharmaceutical formulations failed to produce an appreciable paw-licking response, a higher dose of citric acid (100 mm) at a lower pH of 3.5 produced a paw-licking response similar in duration to 5% formalin (*p* < 0.0001, compared with 30 mm or 100 mm citric acid at pH 5.5; *p* = 0.9961, compared with 5% formalin; Fig. 8*A*). Unlike the ASIC1-dependency of acid-mediated withdrawal reflex (Fig. 2), paw-licking responses induced by either 5% formalin or 100 mm citric acid, pH 3.5, could not be hindered by amiloride (*p* = 0.9483 for formalin, *p* = 0.8607 for citric acid; Fig. 8*B,C*), and paw-licking response induced by 100 mm citric acid, pH 3.5, remained intact in *ASIC1*^−/−^ mice (*p* = 0.7904 compared with wild-type mice; Fig. 8*D*). In the writhing test, intraperitoneal injection of 1% acetic acid at pH 3.5 readily induced episodes of abdominal contraction (*p* < 0.0001, compared with 30 or 100 mm citric acid) and body contortion (*p* < 0.0001, compared with 30 or 100 mm citric acid), whereas intraperitoneal injection of 30 mm citric acid, pH 5.5, induced no writhing response, and 100 mm citric acid, pH 3.5, induced little abdominal contractions and no body contortion (Fig. 9*A,B*). Given previously reported data showing that acetylcholine-mediated writhing response is cholinergic receptor dependent (Collier et al., 1968) and our data showing that acid-induced withdrawal response is ASIC1 dependent (Fig. 2*A*), we next assessed whether acetic-acid-mediated writhing response is also ASIC1 dependent. In contrast to acid-induced withdrawal reflex, acetic-acid-mediated abdominal contraction and body contortion could not be inhibited by amiloride (*p* = 0.1209 for abdominal contraction, *p* = 0.2123 for contortion; Fig. 9*C,D*) and remained intact in *ASIC1*^−/−^ mice (*p* = 0.9294 for abdominal contraction, *p* = 0.2598 for contortion; Fig. 9*E,F*). In summary, citric acid, at a concentration and pH typically used in pharmaceutical formulations, failed to produce a noticeable response in the two conventional rodent models of nociception. Interestingly, although a higher concentration and lower pH of citric acid produced paw-licking responses, and acetic acid produced writhing responses, these acid pain responses were ASIC1 independent.

## Discussion

Early evidence from experiments with human subjects demonstrates that the pain caused by direct infusion of acidic solution can be attenuated by the broad-spectrum ASIC inhibitor amiloride (Ugawa et al., 2002; Jones et al., 2004). To investigate this further, we studied the nociceptive response of mice subjected to intraplantar injection of acidic solutions. As in the aforementioned experiments conducted in human subjects, the nociceptive response caused by intraplantar injection of mildly acidic PBAS in mice was attenuated by amiloride, consistent with the notion that ASICs play a role in this process. Because the physiological functions of different ASIC subtypes are best characterized in mice lacking these receptors, we next repeated the same experiments using *ASIC1*^−/−^ and *ASIC2*^−/−^ mice. Previous studies reported in the literature have shown that *ASIC1*^−/−^ mice are born with impaired hippocampal synaptic plasticity and spatial memory (Wemmie et al., 2002; but see Wu et al., 2013), impaired fear expression and memory (Wemmie et al., 2003; Coryell et al., 2008; Ziemann et al., 2009), exacerbated seizure vulnerability (Ziemann et al., 2008), and mitigated susceptibility to ischemic and inflammatory brain injury (Xiong et al., 2004; Friese et al., 2007; Duan et al., 2011). Additionally, it has been shown that *ASIC2*^−/−^ mice have impaired rapidly adapting mechanical sensation (Price et al., 2000; but see Roza et al., 2004), a normal hearing threshold coupled with increased susceptibility to noise (Peng et al., 2004; Roza et al., 2004), and enhanced visual signal transduction and increased susceptibility to light-induced retinal injury (Ettaiche et al., 2004), and the hippocampal neurons of these mice are shown to have reduced numbers of dendritic spines in the CA1 region (Zha et al., 2009). In the present study, we further demonstrated that the nociceptive response caused by intraplantar acid injection was lost in *ASIC1*^−/−^ but not *ASIC2*^−/−^ mice, suggesting that ASIC1 mediates this response. Notably, ASIC3 was not examined in our study because (1) amiloride, which attenuated the nociceptive response in this study, has been shown to activate ASIC3 at neutral pH and potentiate ASIC3 at mildly acidic pH (Yagi et al., 2006; Li et al., 2011), and (2) *ASIC3*^−/−^ mice, although somewhat resistant to some forms of chronic pain, are previously reported to have increased rather than reduced sensitivity to different forms of acute pain (Chen et al., 2002).

In addition to the ASIC1-mediated acute pain caused by subcutaneous acid injection as reported in this study, there is now ample evidence that ASIC subtypes contribute to different types of pain. *ASIC3*^−/−^ mice but not *ASIC1*^−/−^ mice have reduced vulnerability to acid-induced mechanical hypersensitivity (Price et al., 2001; Sluka et al., 2003) and serotonin-induced acid hypersensitivity (Wang et al., 2013). Following carrageenan-induced muscle inflammation, *ASIC1*^−/−^ mice have impaired primary mechanical hyperalgesia and intact secondary mechanical hyperalgesia (Walder et al., 2010), whereas *ASIC3*^−/−^ mice have intact primary mechanical hyperalgesia but impaired secondary mechanical hyperalgesia (Sluka et al., 2007; Ikeuchi et al., 2008; Yen et al., 2009; Walder et al., 2010). As mentioned earlier, rather than having acute sensory impairment, *ASIC3*^−/−^ mice are found to have increased sensitivity to acute pain caused by acetic acid, heat, and mechanical force (Chen et al., 2002); in comparison, *ASIC1*^−/−^ and *ASIC2*^−/−^ mice, but not *ASIC3*^−/−^ and *TrpV1*^−/−^ mice, have increased sensitivity to acute pain caused by formalin injection (Staniland and McMahon, 2009). Overall, the availability of ASIC knock-out mice has made it possible to delineate the role of each receptor subtype in pain perception and other physiological processes.

Although the past two decades witnessed the discovery of animal toxins that can inhibit ASIC subtypes with greater specificity than amiloride, these toxins generally inhibit more than one combination of ASIC subunits, and their pharmacological properties may be different from those initially observed. For example, the tarantula toxin psalmotoxin-1 (PcTx1) was initially thought to be a specific inhibitor of ASIC1a homomeric receptors (Escoubas et al., 2000), but later studies found that this toxin also inhibits heteromeric receptors composed of ASIC1a/ASIC2a or ASIC1a/ASIC2b (Sherwood et al., 2011; Joeres et al., 2016; Liu et al., 2018). The black mamba venom peptide mambalgin-1 inhibits receptors composed of a large number of ASIC subunit combinations, including homomeric receptors composed of ASIC1a or ASIC1b and heteromeric receptors composed of ASIC1a/ASIC1b, ASIC1a/ASIC2a, and ASIC1a/ASIC2b but has no effect on homomeric receptors composed of ASIC2a or ASIC3 or heteromeric receptors composed of ASIC1a/ASIC3 or ASIC1b/ASIC3 (Diochot et al., 2012). The sea anemone peptide APETx2 was initially thought to be a specific inhibitor of homomeric receptors composed of ASIC3 and heteromeric receptors composed of ASIC1a/ASIC3 and ASIC1b/ASIC3 but has no effect on homomeric receptors composed of ASIC1a, ASIC1b, or ASIC2a or heteromeric receptors composed of ASIC2a/ASIC3 (Diochot et al., 2004). However, more recent evidence demonstrates that this toxin, at a concentration used in previous *in vivo* animal experiments, also inhibits Na_v_1.8, required for signal transduction by primary sensory neurons (Blanchard et al., 2012). This raised the intriguing possibility that the analgesic properties seen with APETx2 may be partly, if not completely, unrelated to its inhibition of ASIC. Therefore, caution needs to be taken when interpreting experimental results based solely on pharmacological agents that inhibit ASIC.

In contrast to protons, citric acid is a small organic acid molecule with distinct pK_a_ values for each of its three carboxylic acid groups; this and other chemical properties have made citric acid a widely used chemical ingredient in many pharmaceutical formulations (Chalgeri and Tan, 1993; Pygall et al., 2009). Indeed, adalimumab (Humira, AbbVie), one of the best-selling pharmaceutical products, had a citrate-buffered acidic formulation, pH 5.2. Like other citrate-containing pharmaceutical products (Frenken et al., 1993; Veys et al., 1998; Yu et al., 1998; Laursen et al., 2006), however, pain at the site of injection was a major complaint of patients receiving injections of adalimumab for the treatment of rheumatoid arthritis and Crohn's disease (Furst et al., 2003; Keystone et al., 2004; van de Putte et al., 2004; Nash et al., 2016). In this study, we found that neutral citrate alone does not cause the nociceptive response seen in mice treated with intraplantar acid injection, but it dramatically potentiates the response when injected simultaneously with acid. To examine this further, we studied the pharmacological action of citrate on HEK cells, which natively expressed human ASIC1, with an ASIC1a-to-ASIC1b transcription ratio similar to that of human primary sensory neurons but not ASIC2 or ASIC3 (Gunthorpe et al., 2001; Yang and Lai, 2020). As in the behavioral data, we found that citrate alone does not activate ASIC1 but strongly potentiates ASIC1-mediated inward current when applied concurrently with acid. Finally, we found that citrate (1) acted extracellularly rather than intracellularly to produce this effect, (2) needs to be present concurrently with acidity to have this effect, and (3) produces this effect chiefly by chelating extracellular Ca^2+^.

Our data on the potentiating effect of citrate-mediated Ca^2+^ removal on acid-mediated response is consistent with the known physiological role of Ca^2+^ on the electrophysiology of ASIC1 and other ion channels of the ASIC family. In particular, Ca^2+^ is thought to be a constitutive open-channel blocker that keeps ASIC3 in the closed state at neutral pH, and when there is a decrease in extracellular pH, protonation of key amino acid residues on the ion channel relieves the channel pore of Ca^2+^ blockade to cause channel opening (Immke and McCleskey, 2003). This model is supported by the finding that removal of extracellular Ca^2+^ sufficiently opens the ASIC3 ion channel without a change in pH (Immke and McCleskey, 2003). In comparison, although Ca^2+^ also modulates the activities of ASIC1a and ASIC1b (Babini et al., 2002; Paukert et al., 2004; Zhang et al., 2006), removal of Ca^2+^ does not efficaciously activate these ion channels without a change in pH (Paukert et al., 2004). Additionally, a recent study found the key amino acid residue required for Ca^2+^-binding to rat ASIC3 and showed that mutation of this residue hindered ASIC3 activation induced by Ca^2+^ removal (Zuo et al., 2018); interestingly, the reverse substitution of the equivalent residue on chicken ASIC1 made the ion channel sensitive to activation induced by Ca^2+^ removal (Zuo et al., 2018). Based on the known dissociation constant of Ca^2+^-bound citric acid (6.0–7.0 × 10^−4^ m; Greenwald, 1938; Joseph, 1946; Walser, 1961), the concentration of free Ca^2+^ in the test solutions used in our electrophysiology experiments would be within the ranges of 42–49 μm, 50–60 μm, 140–160 μm, 560–610 μm, and 1.31–1.34 mm for solutions that contained 30, 25, 10, 3, and 1 mm citrate, respectively. Therefore, given that free Ca^2+^ at a concentration of 100 μm or less has no inhibitory effect on ASIC1 efficacy (Paukert et al., 2004), our test solutions that contained 25 or 30 mm citrate would essentially remove any inhibitory effect of Ca^2+^ on ASIC1.

In conclusion, we show in this study how citric acid causes pain by stimulating and potentiating ASIC1. The acid component of citric acid is required for ASIC1 stimulation, and the citrate component, although on its own does not activate ASIC1, strongly potentiates acid-induced ASIC1 activation by chelating extracellular Ca^2+^ that normally inhibits the receptor. This explains why injections of some pharmaceutical products are so painful when their formulations are only mildly acidic. Although pharmaceutical products can sometimes be reformulated without citrate (as has been the case of Humira Citrate-free), this might not always be a feasible or cost-effective option. To avoid painful injections without the need to eliminate citrate from drug formulations, it might be possible to formulate therapeutic agents with inhibitors of ASIC1 or with supplemented Ca^2+^ ions to saturate ASIC1 in the presence of citrate.
